# COCARDE Study–Cardiac Imaging Phenotype in Patients With COVID-19: Protocol for a Prospective Observational Study

**DOI:** 10.2196/24931

**Published:** 2022-01-06

**Authors:** Olivier Lairez, Virginie Blanchard, Laurent Balardy, Fanny Vardon-Bounes, Stéphanie Cazalbou, Stéphanie Ruiz, Samia Collot, Valérie Houard, Yves Rolland, Jean-Marie Conil, Vincent Minville

**Affiliations:** 1 Department of Cardiology Rangueil University Hospital Toulouse France; 2 Gerontopole of Toulouse Institute of Ageing Toulouse University Hospital Toulouse France; 3 Department of Anesthesiology and Intensive Care Toulouse University Hospital Toulouse France; 4 Department of Radiology Rangueil University Hospital Toulouse France

**Keywords:** COVID-19, SARS-CoV-2, cardiac imaging, echocardiography, cardiac MRI, cardiac imaging, hyperinflammation, inflammation

## Abstract

**Background:**

The effects of SARS-CoV-2 (COVID-19) on the myocardium and their role in the clinical course of infected patients are still unknown. The severity of SARS-CoV-2 is driven by hyperinflammation, and the effects of SARS-CoV-2 on the myocardium may be significant. This study proposes to use bedside observations and biomarkers to characterize the association of COVID-19 with myocardial injury.

**Objective:**

The aim of the study is to describe the myocardial function and its evolution over time in patients infected with SARS-CoV-2 and to investigate the link between inflammation and cardiac injury.

**Methods:**

This prospective, monocentric, observational study enrolled 150 patients with suspected or confirmed SARS-CoV-2 infection at Toulouse University Hospital, Toulouse, France. Patients admitted to the intensive care unit (ICU), regular cardiologic ward, and geriatric ward of our tertiary university hospital were included during the pandemic period. Blood sampling, electrocardiography, echocardiography, and morphometric and demographic data were prospectively collected.

**Results:**

A total of 100 patients were included. The final enrolment day was March 31, 2020, with first report of results at the end of the first quarter of 2021. The first echocardiographic results at admission of 31 patients of the COCARDE-ICU substudy population show that biological myocardial injury in COVID-19 has low functional impact on left ventricular systolic function.

**Conclusions:**

A better understanding of the effects of COVID-19 on myocardial function and its link with inflammation would improve patient follow-up and care.

**Trial Registration:**

Clinicaltrials.gov NCT04358952; https://clinicaltrials.gov/ct2/show/NCT04358952

**International Registered Report Identifier (IRRID):**

DERR1-10.2196/24931

## Introduction

### Background

SARS-CoV-2 infection, the pathogen which is responsible for COVID-19 and which can lead to acute respiratory distress syndrome, is not limited to the pulmonary sphere and has systemic effects that contribute to its significant mortality. The effects of SARS-COV-2 on the myocardium and their role in the clinical course of infected patients are still unknown. Cardiovascular risk factors such as diabetes and hypertension, as well as coronary or cerebral cardiovascular history, have been associated with severe forms of infection [1-3]. In addition, most of the cohort data currently available report biological myocardial injury with troponin increase in about 12% of patients [4-6] with a prevalence ranging from 4% to 31% according to the burden of the disease and the need for resuscitative management [4,7,8]. Myocardial injury is associated with excess mortality, which is particularly prevalent in older adults [7-9]. Individuals at greatest risk of severe disease and mortality are older patients, particularly those with underlying cardiovascular risk factors and chronic conditions.

The pathophysiology of the myocardial involvement of SARS-CoV-2 remains poorly understood despite research flourishing in the field [10]. Some observations suggest an association with the cytokine storm that accompanies the infection [5]. The more or less massive release of proinflammatory cytokines has been linked to the pathophysiology of organ failure that accompanies viral infection [11], further explaining the prognostic role of several biological parameters such as increased C-reactive protein, procalcitonin, D-dimers, creatine phosphokinase, lactate dehydrogenase, lymphopenia, and leukopenia [2,4,12,13]. The cytokine storm is thought to cause an imbalance between myocardial oxygen needs and supply, leading to a type 2 myocardial infarction [14]. However, given the importance of cardiovascular risk factors in severe forms of the disease, a type 1 myocardial infarction [14] via an atheromatous plaque rupture mechanism cannot be excluded [15,16] despite there being a trend of reduced admissions for acute coronary syndrome [17]. Finally, the possibility of a myocardial tropism of SARS-CoV-2 through the myocardial angiotensin-converting enzyme 2 receptors [18] could explain a direct viral infection of the myocardium, which may lead to fulminant myocarditis [19,20]. More generally, the inflammatory mechanisms involved could also affect skeletal muscle tissue and body composition resulting in cardiac cachexia [21].

However, despite there being substantial biological data on cardiac injury during SARS-CoV-2 infection, to date, no data on the functional impact of the infection on the myocardium have been collected.

### Rationale

Based on the observation that cardiac injury—as evidenced by an increase in troponin—is associated with a poor prognosis and that the most severe forms affect older adult patients, the COCARDE study was designed in 2 arms: intensive care unit (COCARDE-ICU) and geriatrics (COCARDE-Geria) with the aim to describe the cardiac imaging phenotype of patients infected with SARS-CoV-2.

### Objectives

The objective of this study is to describe the myocardial function and its evolution during infection in high-risk patients infected with SARS-CoV-2 and to establish the role of inflammation in the cardiac involvement.

## Methods

### Study Design

**Figure 1:**
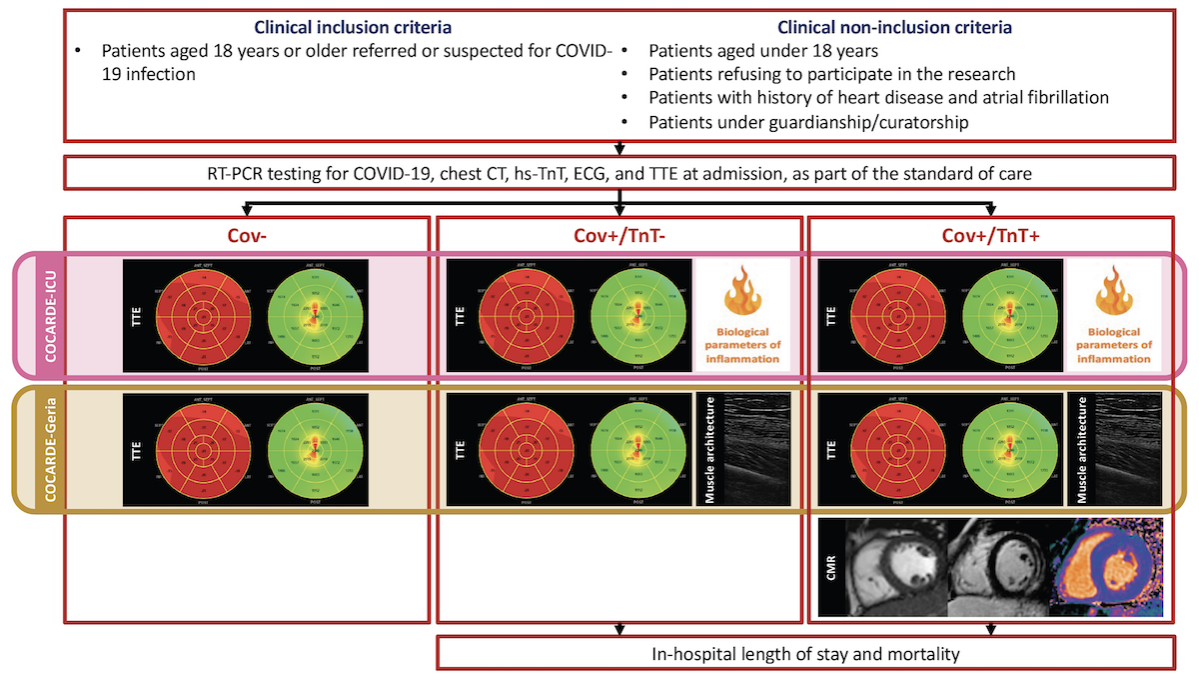
Patient flowchart. CMR: cardiac magnetic resonance; COV–: without SARS-CoV-2 infection; COV+: with SARS-CoV-2 infection; CT: computed tomography; ECG: electrocardiography; hs-TnT: high-sensitivity troponin T; RT-PCR: real-time reverse transcription–polymerase chain reaction; TnT–: without biological cardiac injury; TnT+: with biological cardiac injury; TTE: transthoracic echocardiography.

The COCARDE study is an investigator-initiated, prospective, monocentric, observational study with a planned enrolment of 150 patients at Toulouse University Hospital, Toulouse, France. The patient flowchart is presented in Figure 1.

### Notifications and Registration

The study was considered to as appropriate by the national ethics committees (protocol number ID-RCB: 2020-A00852-37; positive endorsement of the protection of persons committee West IV dated April 8, 2020) and the French Data Protection Agency. The study is registered with Clinicaltrials.gov (NCT04358952). The investigation conforms with the principles outlined in the Declaration of Helsinki. All patients or their dependents have been informed that the data collected can be used for research purposes, and the absence of opposition has been collected for each patient in accordance with French legislation.

### Objectives

Primary and secondary objectives and definitions of the COCARDE study are listed in Textbox 1.

**Primary and secondary objectives and definitions of the COCARDE study.**

**Primary objective**

- Prospectively describe the myocardial function at admission in a population of high-risk patients infected with SARS-CoV-2 compared to a matched population of uninfected patients.

**Primary objective definition**

- Myocardial function assessed by global longitudinal strain and myocardial work indices (global work index and global work efficiency) by speckle-tracking echocardiography.

**Secondary objectives**

- Describe the evolution of myocardial function over time (admission, day 3, day 7, and day 14) in high-risk patients infected with COVID-19.
- Describe the relationship between myocardial function and biological parameters of inflammation in high-risk patients infected with COVID-19.
- Describe the relationship between myocardial function and vastus lateralis muscle architecture in patients over 70 years old infected with COVID-19.
- Describe the myocardial lesion pattern by cardiac magnetic resonance imaging in patients infected with COVID-19 with biological cardiac injury.
- Describe the impact of myocardial function on prognosis in hospitalized patients infected with COVID-19.

**Secondary objectives definitions**

- Myocardial function assessed by global longitudinal strain and myocardial work indices (global work index and global work efficiency) by speckle-tracking echocardiography.
- Biological parameters of inflammation: proinflammatory cytokines, anti-inflammatory cytokines, alarmins, and resolvins.
- Morphometric parameters assessed by muscle architecture to determine muscle thickness, penetration angle, and muscle fiber length of the vastus lateralis using ultrasound [22].
- Myocardial lesion pattern assessed by T2-weighted, T2 mapping, and precontrast T1 mapping imaging. (edema); and early gadolinium enhancement (hyperemia), late gadolinium enhancement, and postcontrast T1 mapping (necrosis/edema) by cardiac magnetic resonance imaging.
- Prognosis assessed by hospital length of stay and in-hospital mortality.

### Patient Population

Following the classification of the SARS-CoV-2 infection as a pandemic by the World Health Organization on March 11, 2020, and following an epidemiological alert issued by the French health authorities on March 14, 2020, the organization of patient reception at our university hospital has been reviewed to allow for the screening and isolation of infected patients. All patients with suspected SARS-CoV-2 infection have undergone collection of specimens from the upper respiratory tract for SARS-CoV-2 testing by real-time reverse transcription–polymerase chain reaction (RT-PCR), chest computed tomography (CT), cardiac biomarkers including values of high-sensitivity troponin T and N-terminal pro b-type natriuretic peptide, electrocardiography, and transthoracic echocardiography at admission as part of the standard of care.

A diagnosis of a SARS-CoV-2 infection should be retained in the presence of an evocative chest CT and a positive RT-PCR for SARS-CoV-2 or an evocative chest CT in the presence of a negative RT-PCR for SARS-CoV-2 after other common respiratory viral and bacterial infections have been ruled out [23]. Biological cardiac injury was defined as blood levels of high-sensitivity troponin T above the 99th percentile upper reference limit.

Patients have been categorized into 3 groups according to the presence of SARS-CoV-2 infection or biological cardiac injury, and the study population has been divided into 2 arms: patients requiring or not requiring the ICU (COCARDE-ICU) and patients over 70 years old not referred to the ICU (COCARDE-Geria).

The serum of patients from the COCARDE-ICU population was collected after centrifugation for a study of inflammatory parameters. Patients from the COCARDE-Geria population were assessed for the muscle architecture of the vastus lateralis.

Patients with biological cardiac injury were referred for cardiac magnetic resonance imaging when suitable.

### Inclusion Criteria

The inclusion criteria for patients are an age 18 years or older and those referred for or suspected of SARS-COV-2 infection.

### Exclusion Criteria

The exclusion criteria for patients are the following: patients aged under 18 years old; patients refusing to participate to the research; patients with a history of heart disease and atrial fibrillation; and patients under guardianship, curatorship, or safeguard of justice.

### Data Collection

The demographic characteristics (age and sex), clinical data (weight, height, symptoms, delay between first symptom and admission, comorbidities, treatments), arterial blood pressure, and laboratory findings for patients during hospitalization were collected from electronic medical records and entered into the database by 2 investigators (VB and VH). Electrocardiography, transthoracic echocardiography, cardiac magnetic resonance imaging, and muscle architecture assessment were performed and analyzed by independent investigators blinded to the clinical characteristics of the patients.

### Biomarkers

Inflammatory pathway parameters were collected for plasma extracted from venous blood sampling into sodium citrate tubes. Blood samples were centrifuged at 3000 g for 5 minutes at room temperature. Superior two-thirds supernatant was recovered and again centrifuged for 5 minutes. The following analyses were then performed in the same laboratory: proinflammatory cytokines, including tumor necrosis factor alpha, interleukin 6 (IL-6), IL-1β, IL-1α, interferon gamma (IFN-γ), IFN-α2, monocyte chemoattractant protein-1 (CCL2), IL-12, IL-17, IL-23, IL-33, and IL-8 (CXCL8); the anti-inflammatory cytokine, IL-10; alarmins, including high-mobility group box protein 1, S100 protein, and DNA; and resolvins, including 18-HETE (18-hydroxyarachidonic acid), 15-HETE, 12-HETE, 17-HDOHE (17-hydroxydocosahexaenoic acid), 5-HETE, 14-HDOHE, eicosapentaenoic acid, docosahexaenoic acid, arachidonic acid, protectin DX, prostaglandin, and thromboxan B2.

### Electrocardiography

Electrocardiography was analyzed for heart rate, rhythm, intraventricular conduction delay, QRS duration, and QT duration.

### Transthoracic Echocardiography and Image Analysis

Transthoracic echocardiography was performed with either a Vivid E95 or Vivid S70 ultrasound system (GE Healthcare) using a 3.5 MHz transducer, which facilitated the archiving of acquisitions for a deferred analysis. Doppler, M-mode, and 2D gray scale echocardiography including the 3 standard apical views (4-, 3-, and 2-chamber) with high frame rates (>60 frames/s); pulsed Doppler transmitral inflow and left ventricular outflow; and the pulsed-Doppler tissue imaging lateral mitral and tricuspid annular velocities were performed for each patient with simultaneous arterial blood pressure recording.

Image analyses were performed offline using EchoPAC V202 software (GE Medical Systems). Two-dimensional and Doppler echocardiography measurements and quantification were performed according to the American Society of Echocardiography and the European Association of Cardiovascular Imaging guidelines [24,25]. The following measurements were collected: diastolic parameters, including peak early and late diastolic mitral inflow velocity, E/A ratio, deceleration time, lateral mitral annular diastolic velocity, peak systolic tricuspid annular velocity, and tricuspid annular plane systolic excursion. All Doppler measurements were made over 3 cardiac cycles and averaged. Left ventricular end-diastolic and -systolic volumes and ejection fraction were measured using the modified biplane Simpson’s method from apical 2- and 4-chamber views. Global longitudinal strain (GLS) was calculated from the average of the segmental strain on a 17-segment model using 2D speckle tracking from grayscale images and the automated function imaging technique from the apical 4-chamber, 3-chamber, and 2-chamber views [26]. Myocardial work was calculated from left ventricular GLS and the estimated left ventricular pressure curve as proposed by Russell et al [27]. The wasted work is defined by the work performed by the myocardium during segmental elongation and the constructive work by the work performed during segmental shortening. During isovolumetric relaxation, this definition is reversed such that myocardial work during shortening is considered wasted work and work during lengthening is considered constructive work. Work efficiency is then calculated as the constructive work divided by the sum of the constructive and the wasted work.

### Muscle Architecture Processing and Analysis

Skeletal muscle ultrasound assessment of the vastus lateralis was performed using a Vivid E95 ultrasound system (GE Healthcare) and a 15 MHz linear probe by acquisition at the lower third of the femur for exploration as described by Aubertin-Leheudre et al [22]. In this procedure, patients sit with hip and knee angled at 90° and with limb muscles relaxed. The probe is positioned perpendicular to the dermal surface of the vastus lateralis muscle and oriented along the median longitudinal plane of the muscle. For this study, 3 sagittal ultrasounds of the vastus lateralis were then digitized and images analyzed offline using EchoPAC V202 software (GE Medical Systems) to determine muscle thickness (distance from the superior and deepest aponeurosis at the greatest distance), penetration angle (angle of insertion of the bundle of muscle fibers into the deep aponeurosis), and muscle fiber length (length of the fascicle between the superior and deep aponeurosis).

### Cardiac Magnetic Resonance Protocol and Imaging Analysis

Cardiac magnetic resonance was performed in breath-hold mode with the use of a 1.5-T Ingenia Ambition X magnetic resonance imaging system (Philips Medical Systems) using a 32-element phased-array cardiac coil with cardiac gating in accordance with the recommendations of the Society for Cardiovascular Magnetic Resonance endorsed by the European Association for Cardiovascular Imaging [28]. Following scout imaging, balanced steady-state free precession breath-hold images were acquired with a slice thickness of 6 mm (long-axis and 4-chamber views) or 8 mm (contiguous short-axis views with no gap between slices from the atrioventricular ring to the apex). Subsequently, standard sequences for T2-weighted, T2 mapping, and precontrast T1 mapping images were obtained in the short axis, through the basal, midcavity, and apical slices, and then early and late gadolinium enhancement images were obtained in the long-axis, 4-chamber, and short-axis orientations 10 minutes after the injection of 0.2 mmol/kg of gadolinium dimenglumine (Magnevist) using a phase-sensitive inversion recovery spoiled gradient echo sequence. Postcontrast T1 mapping was obtained in the short axis, through the basal, midcavity, and apical slices.

Image analysis was performed using the clinically available imaging software workstation ViewForum (Philips Medical Systems). The endocardial border was outlined on the short-axis cine images on the right and left ventricles, in systole and diastole, from the base to the apex to calculate volumes and ejection fractions. T2 and pre- and postcontrast T1 mapping measurements were performed on motion-corrected maps to cover the entire myocardium in the short axis, and 6 separate segments where both mean and maximum values were noted. The extent and pattern of late gadolinium enhancement were assessed by a planimeter on the short-axis contrast images and confirmed on an orthogonal view (either long-axis or 4-chamber) with the use of an image intensity level ≥2 SDs above the mean of the remote myocardium to define late gadolinium enhancement. The 17-segment model was used to localize late gadolinium enhancement within the left ventricle.

### Sample Size

This is a descriptive pilot study. As there is no data on the topic, to date, the number of patients included is 50 per group. The group allocation is as follows: 50 patients without SARS-CoV-2 infection (Cov–), 25 in the COCARDE-ICU substudy and 25 in the COCARDE-Geria substudy; 50 patients with SARS-COV-2 infection without biological cardiac injury (Cov+/TnT–), 25 in the COCARDE-ICU substudy and 25 in the COCARDE-Geria substudy; and 50 patients with SARS-COV-2 infection with biological cardiac injury (Cov+/TnT+), 25 in the COCARDE-ICU substudy and 25 in the COCARDE-Geria substudy.

### Timeline for Myocardial Function Monitoring

**Figure 2:**
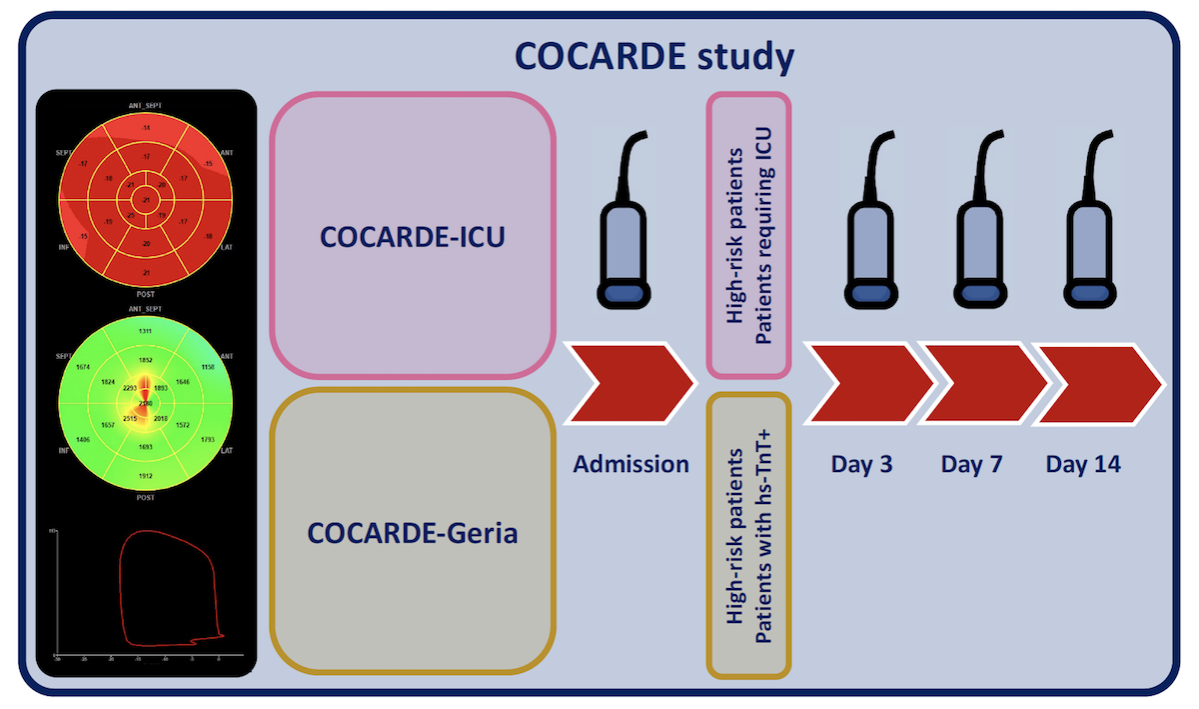
Timeline for myocardial function monitoring. ICU: intensive care unit; hs-TNT: high-sensitivity troponin T.

The timeline for myocardial function monitoring is presented in Figure 2.

### Statistical Analysis

Continuous variables have been tested for normal distribution using the Kolmogorov-Smirnov test and are expressed as mean and SD. Laboratory findings were not normally distributed, and the results will be, therefore, presented as medians with IQR. Nominal values will be expressed as numbers and percentages. The study population was categorized into 3 groups: Cov–, Cov+/TnT–, and Cov+/TnT+. Group comparisons have been made using nonparametric Kruskal-Wallis tests or analysis for variance for continuous variables and χ2 test for categorical variables, with Bonferroni corrections being used for multiple comparisons. Logistic regression models and classification regression trees were used to identify predictors of myocardial dysfunction at admission. Differences are being considered statistically significant for *P* values <.05. All analyses were performed using SPSS version 20 software (IBM Corp).

## Results

A total of 100 patients were included. The final enrolment day was March 31, 2021, with first report of results at the end of the first quarter of 2021.

The first results of the study presenting the echocardiographic phenotype at admission of 31 patients of the COCARDE-ICU substudy population have been published, showing that biological myocardial injury in COVID 19 has low functional impact on left ventricular systolic function [29]. Longitudinal data are being collected and the COCARDE-Geria substudy data are being analyzed.

## Discussion

The COCARDE study is the first study to date to propose a cardiac imaging phenotyping of patients infected with SARS-CoV-2. Data on myocardial injury in these patients are scarce and limited to biological parameters of myocardial injury. Although sensitive, these parameters do not prejudge the mechanism and functional impact on the myocardium. Troponin is a marker of myocardial injury, including, but not limited to, myocardial infarction or myocarditis, and the clinical relevance of this distinction has never been clear and is even less clear in the context of the SARS-COV-2 infection, which causes a plethora of ischemic and nonischemic origins of myocardial lesions [30]. However, to date, most of the data regarding SARS-CoV-2–associated cardiovascular complications are anecdotal and in the absence of systematic studies [31].

The COCARDE study will be able to describe myocardial function assessed by GLS and myocardial work indices in a population patients infected with SARS-COV-2. Furthermore, the study will be able to describe the evolution over the time of myocardial function among the high-risk patients in this population (ie, patients hospitalized in the intensive care unit and older patients with biological cardiac injury).

This study will help in understanding the impact of the kinetics of inflammatory parameters on myocardial function during infection. The magnetic resonance imaging data will allow for the differentiation between direct myocardial involvement by the inflammatory process and indirect vascular involvement, regardless of whether it is a type 1 or type 2 myocardial infarction.

The COCARDE study has the limitations associated with single-site and limited-sample studies. Therefore, our patients may not represent patients admitted to other facilities for SARS-CoV-2 management. However, the study is being carried out in a tertiary care teaching hospital to which the majority of patients of the suburban area are referred. Moreover, the selection of high-risk patients (ie, patients hospitalized in the intensive care unit and older patients) provides a choice sample to help better understand myocardial injury in the population of patients infected with SARS-CoV-2.

Given the severity of illness and the primary focus on urgently managing infection and respiratory failure, it is understandable that not all patients have complete cardiac data, such as electrocardiography, and that information from more sophisticated cardiac testing, such as echocardiography, coronary angiography, and magnetic resonance imaging, are not available [15]. To date, there are no data on the relationship between myocardial function and biological parameters of inflammatory activation pathways in patients infected with SARS-CoV-2. This study proposes to use bedside observations and biomarkers to characterize the association SARS-COV-2 with myocardial injury.

## Abbreviations

Cov–: without SARS-CoV-2 infection
Cov+: with SARS-CoV-2 infection
CT: computed tomography
GLS: global longitudinal strain
HDOHE: hydroxydocosahexaenoic acid
HETE: hydroxyarachidonic acid
IFN: interferon
IL: interleukin
RT-PCR: real-time reverse transcription–polymerase chain reaction
TnT–: without biological cardiac injury
TnT+: with biological cardiac injury
TTE: transthoracic echocardiography
